# Accurate real space iterative reconstruction (RESIRE) algorithm for tomography

**DOI:** 10.1038/s41598-023-31124-7

**Published:** 2023-04-06

**Authors:** Minh Pham, Yakun Yuan, Arjun Rana, Stanley Osher, Jianwei Miao

**Affiliations:** 1 Department of Mathematics, University of California, Los Angeles, CA 90095 USA; 2 Department of Physics and Astronomy, California NanoSystems Institute, University of California, Los Angeles, CA 90095 USA; 3 Present Address: Zhangjiang Institute for Advanced Study, Shanghai Jiao Tong University, Shanghai, 200240 China

**Keywords:** Applied physics, Mathematics and computing, Nanoscale materials, Imaging techniques

## Abstract

Tomography has made a revolutionary impact on the physical, biological and medical sciences. The mathematical foundation of tomography is to reconstruct a three-dimensional (3D) object from a set of two-dimensional (2D) projections. As the number of projections that can be measured from a sample is usually limited by the tolerable radiation dose and/or the geometric constraint on the tilt range, a main challenge in tomography is to achieve the best possible 3D reconstruction from a limited number of projections with noise. Over the years, a number of tomographic reconstruction methods have been developed including direct inversion, real-space, and Fourier-based iterative algorithms. Here, we report the development of a real-space iterative reconstruction (RESIRE) algorithm for accurate tomographic reconstruction. RESIRE iterates between the update of a reconstructed 3D object and the measured projections using a forward and back projection step. The forward projection step is implemented by the Fourier slice theorem or the Radon transform, and the back projection step by a linear transformation. Our numerical and experimental results demonstrate that RESIRE performs more accurate 3D reconstructions than other existing tomographic algorithms, when there are a limited number of projections with noise. Furthermore, RESIRE can be used to reconstruct the 3D structure of extended objects as demonstrated by the determination of the 3D atomic structure of an amorphous Ta thin film. We expect that RESIRE can be widely employed in the tomography applications in different fields. Finally, to make the method accessible to the general user community, the MATLAB source code of RESIRE and all the simulated and experimental data are available at https://zenodo.org/record/7273314.

## Introduction

Tomography has had a radical impact on diverse fields ranging from the 3D determination of the individual atoms in matter to the diagnosis of disease in medicine^1–4^. In the physical sciences, atomic electron tomography (AET) has been developed to resolve the atomic structure of crystal defects and amorphous materials in unprecedented 3D detail^1,3,5–10^. In the biological sciences, cryo-electro nmicroscopy has become a powerful tool for 3D structural determination of macromolecules with identical or similar conformations at near-atomic resolution^2,11–14^. For pleomorphic biological structures, cryo-electron tomography is a method of choice with a resolution in the range of 2–5 nm^15,16^. Tomography has also been combined with coherent diffractive imaging methods^17–19^ to perform quantitative 3D imaging of thick samples with a resolution of tens of nanometers^20–29^. In medicine, computed tomography has been routinely used as a diagnostic imaging procedure^4^. Although the applications of tomography are wide and diverse, a central problem associated with its mathematical and experimental implementation is similar, that is, how to accurately reconstruct a 3D structure from noisy and incomplete projection data^30^. When there are a large number of projections with low noise and no missing data, a direct inversion technique, named filtered back-projection (FBP)^2,4,30^, is accurate and fast. However, for most applications, there are usually missing data and high noise due to the radiation damage to the samples and/or the geometric constraint on the tilt range. To deal with these issues, real-space iterative algorithms have been developed such as algebraic reconstruction technique (ART)^30,31^, simultaneous algebraic reconstruction technique (SART)^32^ and simultaneous iterative reconstruction technique (SIRT)^33,34^. These algorithms minimize the difference between measured and calculated projections using least-square optimization, which can be implemented in parallel computing with a fast running time. Although ART, SIRT and SART usually outperform FBP, the forward and back projection steps in the real-space iterative algorithms are based on mathematical approximations that are not accurate enough to produce high-quality reconstructions. Fourier-based iterative algorithms have been developed to overcome this accuracy limitation, such as equal slope tomography (EST), generalized Fourier iterative reconstruction (GENFIRE) and others^8,35–39^, which rely on the Fourier transform and the inversion as the accurate forward and back projection steps, respectively. Numerical simulation and experimental results have demonstrated that EST and GENFIRE perform better than FBP, ART, SIRT and SART when there are only a limited number of projections^8,35,36,40–43^. EST and GENFIRE have also played an essential role in the development of AET for the 3D determination of crystal defects at the single-atom level^1,3,5–10,44^. Albeit powerful, Fourier-based iterative algorithms require a large memory to assemble a 3D Fourier array in each reconstruction and are also computationally expensive due to the difficulty in implementing parallel computing^9,35,36^. Here, we report the development of RESIRE for accurate tomographic reconstruction. Compared with real-space iterative algorithms, RESIRE uses the Fourier slice theorem or the Radon transform as the forward projection and a linear transformation as the back projection, both of which are accurate. Compared with EST and GENFIRE, RESIRE is a real-space iterative algorithm and can be implemented in parallel computing with a fast running time. Our numerical simulation and experimental results indicate that RESIRE outperforms all existing algorithms for accurate tomographic reconstruction. Furthermore, RESIRE has been used by AET to determine the 3D atomic structure of amorphous materials for the first time^45,46^.

## Methods

### Mathematical foundation of RESIRE

The tomographic reconstruction can be formulated as a least-square optimization problem which minimizes the following sum of squared errors (SSE)

1 $$\begin{aligned} \min _O \; \varepsilon (O) = \frac{1}{2} \sum _{\theta } \Vert \Pi _{\theta } (O) - b_{\theta } \Vert ^2_F \end{aligned}$$

where $\{b_{\theta }\}_{\theta }$ and $\{\theta \}_{\theta }$ are the projections and their corresponding tilt angles measured from a 3D object O. We denote that $\Pi _{\theta }$ is a linear projection operator with respect to the tilt angle $\theta$. For simplicity, we represent Eq. (1) with a single tilt axis, but RESIRE works as well for multiple tilt axis case with three Euler angles. The error metric $\varepsilon$ can be decomposed into a sum of $\varepsilon _{\theta }$ which is the error metric for each projection $b_{\theta }$.

2 $$\begin{aligned} \varepsilon _{\theta }(O) = \frac{1}{2} \sum _{x,y} | \Pi _{\theta }(O) \{x,y\} - b_{\theta } \{x,y\} |^2 \end{aligned}$$

The gradient of the error metric $\varepsilon$ was computed with respect to the object *O* using the calculus of variation method^47^. The following derivation is a discrete version of the continuous problem. For a voxel with a 3D coordinate $\{u,v,w\}$, the gradient is calculated by:

3 $$\begin{aligned} \frac{\partial \varepsilon _{\theta } }{\partial O \{ u,v,w \} }&= \sum _{x,y} \Big ( \Pi _{\theta } (O) \{x,y\} - b_{\theta } \{x,y\} \Big ) \frac{ \partial }{ \partial O \{ u,v,w \} } \sum _z O \left\{ R_{\theta } \begin{bmatrix} x\\ y\\ z \end{bmatrix} \right\} \\ &= \Pi _{\theta } (O) \{x,y\} - b_{\theta } \{x,y\} \quad \text{where}\quad \begin{bmatrix} u \\ v \\ w \end{bmatrix} = R_{\theta } \begin{bmatrix} x \\ y \\ z \end{bmatrix} \quad \text{for some}\, z \end{aligned}$$

where $R_{\theta }$ is the rotation matrix. The first line of Eq. (3) is obtained by the vanilla chain rule and the second line is based on an assumption that each voxel of the object is independent from each other, that is, $\partial O \{x,y,z \} / \partial O \{u,v,w \} = 1$ if $\{x,y,z\} = \{u,v,w\}$ and 0 otherwise. As the transpose of a rotation matrix is its inverse, we derive the following transformation

4 $$\begin{aligned} \begin{bmatrix} x\\ y \end{bmatrix} = \begin{bmatrix} R_{1,1} &{} R_{2,1} \\ R_{1,2} &{} R_{2,2} \end{bmatrix} \begin{bmatrix} u\\ v \end{bmatrix} + \begin{bmatrix} R_{3,1} \\ R_{3,2} \end{bmatrix} w \end{aligned}$$

where $R_{i,j}$ is the (*i*, *j*)th element of the rotation matrix $R_{\theta }$, and all the voxels $O\{u,v,w\}$ are on a Cartesian grid (integer numbers) but the coordinates $\{x,y\}$ are not.

Using Eqs. (3)–(4), RESIRE iterates between an updated object and the measured projections with each iteration consisting of a forward and back projection step. In the forward projection step, the projections, $\Pi _{\theta }(O)$, are calculated from the object O of the current iteration using one of the two approaches: the Fourier slice theorem (FST) or the Radon transform. The FST approach first pads zeros to the object and then computes its oversampled Fourier transform, where padding zeros in real space is equivalent to oversampling in reciprocal space and can improve the accuracy of the approach^48^. 2D projections can then be calculated by taking the inverse Fourier transform of the corresponding 2D slices through the origin of the oversampled 3D Fourier transform. Alternatively, the projections, $\Pi _{\theta }(O)$, can be computed from object O via the Radon transform^49,50^. Each voxel of the object is divided into equally sub-voxels, each of which is independently projected along specific tilt directions to calculate 2D projections with sub-pixel precision. The finer the sub-pixel is, the better the precision is, but at the expense of more computational power. In the “back projection” step, the measured projections are subtracted from the forward projections to obtain the differences $\Pi _{\theta }(O) - b_{\theta }$. The gradient of the error metric $\varepsilon _{\theta } (O)$ is computed by applying $\Pi ^T$ on this difference $\Pi _{\theta }(O) - b_{\theta }$. Equation (4) shows how to “back-project” a 2D image to 3D domain. The “back-projection” is in fact a linear transformation which includes a rotation and a translation, that is, each (*u*, *v*) slice of the gradient is a linear transformation of the difference. In addition, the (*u*, *v*) slices where $w\ne 0$ corresponds to the translation of the (*u*, *v*) slice where $w=0$. The amount of the shift is the zero order term $[R_{3,1} \;\; R_{3,2}]^T w$ in Eq. (4).

#### *Remarks*

1. Regarding performance, the FST requires the oversampling ratio OR to be greater than 3; however, OR larger than 4 makes little or no further improvement. To balance the trade-off between performance and efficiency, we choose $OR=3$ in all our experiments.
2. The “forward projection” using the FST and the Radon transform are slightly different. While the FST produces smoothed images, the Radon transform constructs sharper figures. Based on specific applications, users can choose either the FST or the Radon transform to perform the forward projection step.
3. Although the FST has been commonly used for 3D volume assembly in single particle cryo-EM^38,39^ via a software package named RELION, the limitation is the memory usage and the interpolation in the Fourier domain. RELION uses a statistical approach and works well in the case of numerous measurements. But when the measurements are limited, such as AET, which only has around 50–120 projections in a range of $[-70,70]$ degrees, tomography reconstruction becomes an ill-posed problem. Real-space constraints and regularizers can help to stabilize the reconstruction. Thus, one can further improve the Radon transform-based RESIRE by iteratively applying sparse and Tikhonov regularizes similar to the optimization techniques, such as Alternating Direction Methods of Multiplier^51^ (ADMM), Douglas Rachford^52^ (DR), Primal-Dual Hybrid Gradient^53^ (PDHG), and Gradient Projection Method^54^ (GPM).

### RESIRE convergence

To prove the convergence of RESIRE, we need to find the Lipchitz constant *L* of the gradient that satisfies the inequality

5 $$\begin{aligned} \big \Vert \nabla \varepsilon (O_1) - \nabla \varepsilon (O_2) \big \Vert \le L \big \Vert O_1 - O_2 \big \Vert \quad \forall \; O_1, \, O_2 \end{aligned}$$

The Lipchitz property by Eq. (5) will guarantee that RESIRE converges with the step size 1/*L*. When applying the inequality to RESIRE, we first assume *y* is the single tilt axis and reduce the analysis to the 2D case where the reconstruction is a 2D array of size $N_z \times N_x$. For simplicity, let us assume that it is a square array with $N_x = N_z$, and the object has a compact support with a circle shape of diameter $N_z$ and the center coinciding with the origin of the reconstruction. We then vectorize *x* by stacking its columns into a vector, i.e. $x \in {\mathbb {R}}^{N_z N_x \times 1}$. Next we decompose the projection operator $\Pi _{\theta } \in {\mathbb {R}}^{N_x \times N_z N_x}$ into a product of two operators $S \in {\mathbb {R}}^{N_x \times N_z N_x}$ and $P_{\theta } \in {\mathbb {R}}^{N_zN_x \times N_z N_x}$, i.e. $\Pi _{\theta } = S P_{\theta }$ where $P_{\theta }$ is a rotation operator for angle $\theta$, and *S* is projection operator along the *z* axis:

$$\begin{aligned} S = \begin{bmatrix} \textbf{1}^T &{} 0 &{}\ldots &{} 0 \\ 0 &{} \textbf{1}^T &{}\ldots &{} 0 \\ \vdots &{} \vdots &{} \ddots &{} \vdots \\ 0 &{} 0 &{} \ldots &{} \textbf{1}^T \end{bmatrix} \end{aligned}$$

where $\textbf{1} \in {\mathbb {R}}^{N_z \times 1}$ is a vector of all ones. As all the elements of $P_{\theta }$ are in range [0, 1] and each row of $P_{\theta }$ is summed to 1, the rotation operator $P_{\theta }$ is non-expansive, that is, $\Vert P_{\theta }^T \Vert = \Vert P_{\theta }\Vert \le 1$ where $\Vert .\Vert$ is the $l_2$ induced matrix norm. Furthermore, the rotation of an object must be an orthogonal operator, i.e. $P_{\theta }^T P_{\theta } = I$. Since we approximate the rotation by linear interpolation, we have $P_{\theta }^T P_{\theta } \preceq I$, implying that $I - P_{\theta }^T P_{\theta }$ is semi-positive definite. Based on the above analysis, we derive the following inequalities

6 $$\begin{aligned} \big \Vert \nabla \varepsilon _{\theta }(O_1) - \nabla \varepsilon _{\theta }(O_2) \big \Vert&= \big \Vert P_{\theta }^T S^T S P_{\theta } ( O_1 - O_2) \big \Vert \le \big \Vert P_{\theta }^T S^T S P_{\theta } \big \Vert \; \big \Vert O_1 - O_2 \big \Vert \nonumber \\&\le \big \Vert P_{\theta }^T \big \Vert \; \big \Vert S^T S \big \Vert \; \big \Vert P_{\theta } \big \Vert \; \big \Vert O_1 - O_2 \big \Vert \end{aligned}$$

where all the inequalities are obtained by the triangle inequality. $S^T S \in {\mathbb {R}}^{N_zN_x \times N_z N_x}$ is a block-diagonal matrix, containing $N_x$ identical blocks $\mathbf{1 \, 1}^T \in {\mathbb {R}}^{N_z \times N_z}$ which is a rank-one matrix with all elements equal to one. Since $\mathbf{1 \, 1}^T$ has exactly one non-zero eigenvalue $\lambda = N_z$, it results in $\Vert S^TS\Vert = N_z$. Since $P_{\theta }$ is non-expansive and $\Vert S^TS\Vert =N_z$, we approximate the Lipschitz constant

7 $$\begin{aligned} \big \Vert \nabla \varepsilon _{\theta }(O_1) - \nabla \varepsilon _{\theta }(O_2) \big \Vert \le N_z \big \Vert O_1 - O_2 \big \Vert \end{aligned}$$

This inequality is an important result of the step size analysis, showing that $\nabla \varepsilon _{\theta }$ is $N_z$-Lipshitz. Since there are *n* projections contributing to the gradient, the Lipschitz constant increases by a factor of *n*, that is, the accumulated Lipchitz constant becomes $L = nN_z$. The gradient descent step size is determined by $t/(nN_z)$ where $t\in (0,1]$ is the normalized step size. Although our analysis assumes $N_x = N_z$, in practice we usually have $N_z \le N_x$. Since Eq. (7) shows the upper bound of the Lipchitz constant, the same step size *t* is still applicable when $N_z$ is not too small relative to $N_x$. Furthermore, we can choose larger *t* when the object is sparse. Our experimental results show that RESIRE converges well with $t=2$. This *t* value has been chosen for all our experimental results reported in this paper. Finally, we derive the iterative equation of RESIRE:

8 $$\begin{aligned} O^{k+1}\{u,v,w\} = O^k \{u,v,w\} - \frac{t}{n N_z} \sum _{\theta }\Big ( \Pi _{\theta } (O^k) \{x,y\} - b_{\theta }\{x,y\} \Big ) \end{aligned}$$

where the superscript *k* represents the *k*th iteration. Having shown that $\nabla \epsilon$ is Lipchitz continuous with constant $L = n N_z$, we can conclude that RESIRE with a fixed step size $s = t/(n N_z) \le 1/L$ after k iterations will yield a solution $O^k$ which satisfies:

9 $$\begin{aligned} \epsilon (O^k) - \epsilon (O^*) \le \frac{ \Vert O^0 - O^* \Vert ^2 }{2 s k} \end{aligned}$$

where $\epsilon (O^*)$ and $O^*$ are the optimal error value and solution, respectively. In addition, Eq. (9) shows that the sequences $\{O^k\}_k$ produced by the iteration in the Eq. (8) will converge to the optimal solution $O^*$ in the first order where the rate of convergence is $O(\frac{1}{k})$. The proof can be found elsewhere^55^.

Although the above analysis focuses on the single axis rotation case, RESIRE is generalized to the multiple axis rotation reconstructions. For the general case, we use the three Euler angles $(\phi ,\theta ,\psi )$ to describe the orientation of a 3D object, corresponding to the rotations around the z, y, and x-axes, respectively. The composed rotation matrix is $R_{\{\phi ,\theta ,\psi \}} = Z_{\phi } Y_{\theta } X_{\psi }$ where $Z_{\phi }$, $Y_{\theta }$ and $X_{\psi }$ are the rotation matrices around the z, y, and x-axes, respectively. By replacing $R_{\theta }$ with $R_{\phi ,\theta ,\psi }$ in Eq. (3), RESIRE can be used for the general rotation case.

***RESIRE pseudocode***

The pseudocode of the RESIRE algorithm is described below, which can be implemented with parallel computing with a fast running time.
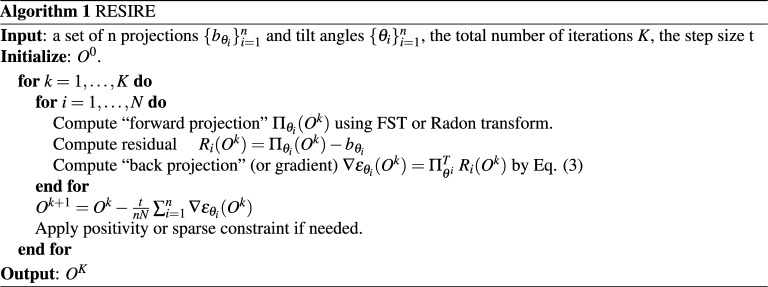


**Figure 1:**
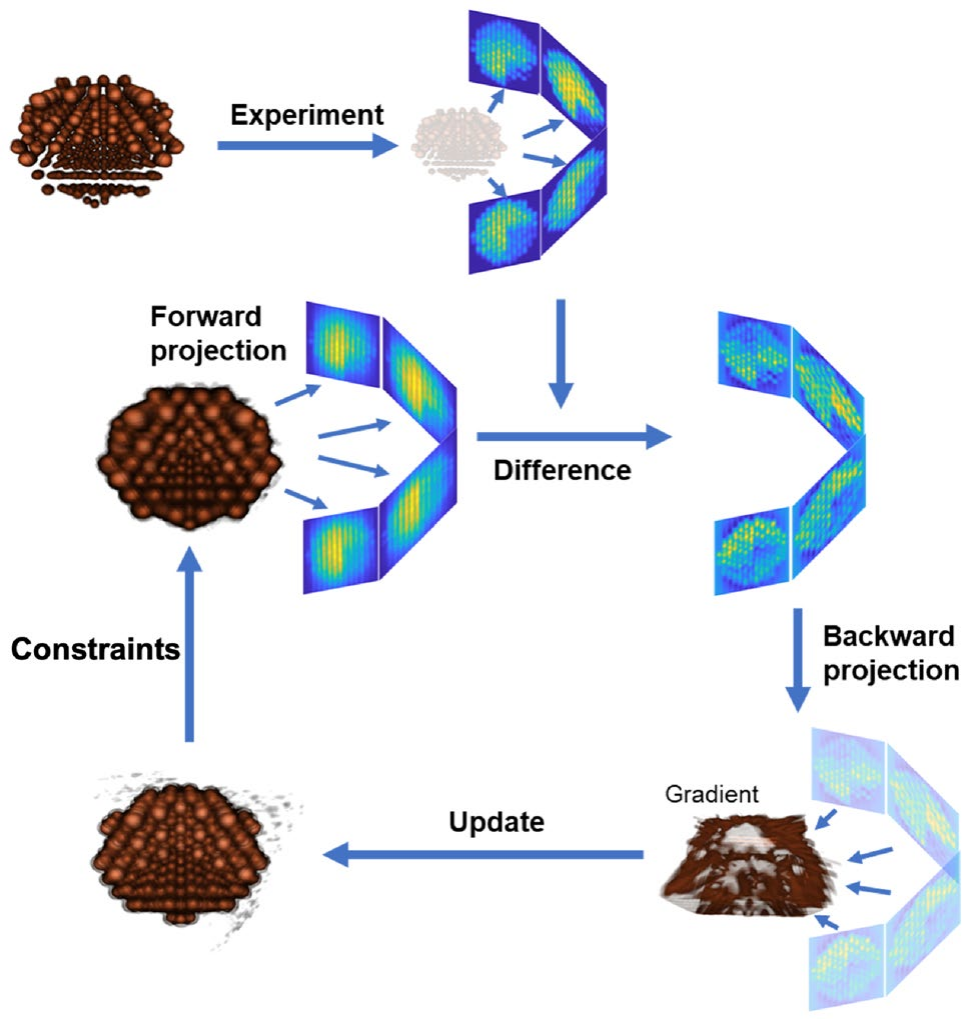
Schematic layout of the RESIRE algorithm. RESIRE first calculates the forward projections of an arbitrary initial object using FST or the Radon transform. The differences between the calculated and measured projections are back-projected to yield the gradient. The initial object is then updated using the gradient. Optional constraints such as positivity and support can be enforced for better convergence and accuracy. The updated object is used for the next iteration. Usually, after several hundreds of iterations, the algorithm converges.

Figure 1 shows the schematic layout of the RESIRE algorithm. To reconstruct a 3D object from given experimental projections, RESIRE minimizes the L2-norm metric using gradient descent. Each iteration of the algorithm includes four steps:

1. RESIRE first computes the forward projections $\Pi _{\theta _i}(O^k)$ of an arbitrary initial object using FST or the Radon transform, where an empty object is often used.
2. The second step computes the differences $R_i (O^k) = \Pi _{\theta _i}(O^k) - b_{\theta _i}$ between the calculated and experimental projections
3. The third step back-projects the difference to 3D real space, yielding the gradient of the object: $\nabla \varepsilon _{\theta _i}(O^k) = \Pi _{\theta ^i}^T \; R_i(O^k)$
4. Lastly, the initial object is updated using the gradient: $O^{k+1} = O^k - \frac{t}{nN} \sum _{i=1}^n \nabla \varepsilon _{\theta _i}(O^k)$. Optional real-space constraints, such as positivity and support, if applicable, are enforced in RESIRE for better convergence and accuracy.

Repeat these four steps until the algorithm converges or the *L*2-norm error does not change. Typically, RESIRE reconstructs high-quality object with sufficiently optimized error metrics after several hundreds of iterations.

### Angular refinement

In many tomography experiments, the measured tilt angles are not always accurate due to instrument misalignment, motor slipping, beam-induced motion, vibration, thermal effects, and/or software error. RESIRE implements the following angular refinement procedure to improve the tilt angle accuracy. Using the current tilt angles, an initial 3D object is reconstructed by RESIRE. For each *j*th measured projection, a series of 2D projections are calculated from the 3D object by varying the three Euler angles $\phi \in [ \phi _j - \delta _{\phi }, \phi _j + \delta _{\phi } ]$, $\theta \in [ \theta _j - \delta _{\theta }, \theta _j + \delta _{\theta } ]$, and $\psi \in [ \psi _j - \delta _{\psi }, \psi _j + \delta _{\psi } ]$ are the current tilt angles. Since our angular refinement uses brutal force, we suggest the searching range is $\pm 3^{\circ }$ within the initial angles for the sake of efficiency, i.e. $\delta _{\phi }=\delta _{\theta }=\delta _{\psi }=3^{\circ }$. Each calculated 2D projection is then compared with the corresponding measured projection via an error metric. The three Euler angles with the smallest R-factor are recorded as the new angles for the *j*th measured projection. After repeating this step for all the measured projections, a new 3D object is reconstructed using the new angles. The refinement procedure is repeated until no further improvement can be made. This angular refinement procedure can improve the tilt angle accuracy and the quality of the 3D reconstruction as demonstrated with numerical simulation and experimental data^36^.

When working with other imaging sciences, such as single-particle, one can consider other refinement processes, such as the EMC algorithm^56,57^. There is a potential to combine RESIRE with such spatial refinements.

## Results

### RESIRE reconstruction of a simulated biological vesicle

**Figure 2:**
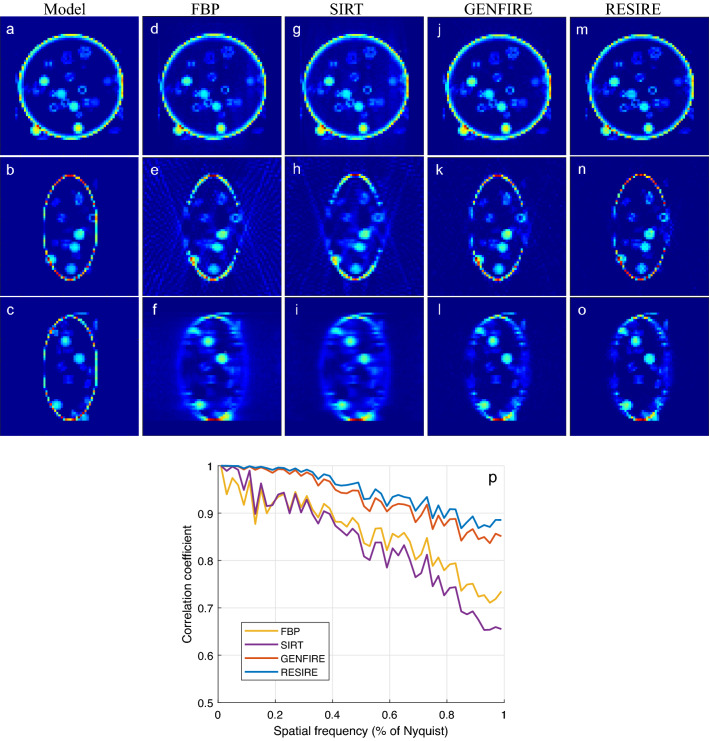
3D reconstruction of a simulated biological vesicle. (**a**–**c**) Three 10-voxel-thick central slices of the vesicle model in the XY, XZ and YZ planes, respectively. The corresponding three reconstructed slices with FBP (**d**–**f**), SIRT (**g**–**i**), GENFIRE (**j**–**l**), and RESIRE (**m**–**o**), where the missing wedge direction is along the z-axis. (**p**) The FSC between the model and the reconstructions showing that RESIRE produces a better reconstruction than FBP, SIRT, and GENFIRE at all spatial frequencies.

A $64\times 64 \times 64$ voxel model of a biological vesicle (Fig. 2a–c) is used in this test to demonstrate the performance of RESIRE relative to the other tomographic reconstruction methods. In the simulation, the tilt axis is the y-axis and the other two Euler angles $\phi$ and $\psi$ are set to zero. When there is enough measurement and no missing wedge, the performance of all Tomography algorithms are all great; hence we omit this test. Instead, we focus on the missing wedge case and large angular increment. The missing wedge problem is believed to be more severe than the coarser tilt angles and causes trouble to Tomographic reconstructions. A tilt series of 41 projections are calculated from the 3D model with a tilt range of $\pm 70^{\circ }$ and an angular increment of $3.5^{\circ }$. Poisson noise is added to each projection to make the simulation data more realistic. Reconstructions are performed using FBP, SIRT GENFIRE and RESIRE. To monitor the convergence, an R-factor $R_F$ is used as an error metric to quantify the difference between the calculated and measured projections.

10 $$\begin{aligned} R_F = \frac{1}{n} \sum _{\theta } \; \left[ \sum _{x,y} |\Pi _{\theta } (O )\{x,y\} | - b_{\theta } \{x,y\} | \Big / \sum _{x,y} | b_{\theta }\{x,y\} | \right] \end{aligned}$$

All the iterative algorithms RESIRE, GENFIRE and SIRT are run with 150 iterations. ASTRA Toolbox^58,59^ is used to perform the SIRT reconstruction and IMOD^60^ is used for FBP. The R-factors are calculated to be 11.7, 23.9, 12.9 and 9.08% for FBP, SIRT GENFIRE, and RESIRE, respectively. By minimizing the least square through iteration, RESIRE and SIRT obtain lower $R_F$ than FBP and GENFIRE. FBP has the largest $R_F$ because it is a direct inversion method. Although its $R_F$ is slightly larger, GENFIRE produces a more accurate reconstruction than SIRT as demonstrated below.

Figure 2 show 10-voxel-thick central slices of the 3D reconstructions in the XY (top row), XZ (middle row) and YZ (bottom row) planes by FBP, SIRT, GENFIRE, and RESIRE, respectively, where the z-axis is the missing wedge direction. The XY central slices from all four methods show a good agreement with the model. Although the missing wedge effects are present in all the four reconstructions of the XZ and YZ slices, RESIRE exhibits less peripheral noise, more easily detectable boundaries and reduces missing wedge effects than FBP, SIRT, and GENFIRE.

To quantify the results, we calculate the Fourier shell correlation (FSC) between the model and each reconstruction. Figure 2p confirms that that RESIRE outperforms the other three methods at all spatial frequencies.

Next, we perform the angular refinement test with the same simulation data, deviating the tilt angles by adding Gaussian noise with a normal distribution (standard deviation equals 1.0) to the ground truth $\theta$ angles. The angular refinement starts with these initial guesses and searches for optimal values in the range of $\pm 3^{\circ }$ within the initial ones. After a couple of rounds of refinement, the Root Mean Square Error (RMSE) between the ground truth angles and the refined ones is reduced from 1.00 to 0.16. The 3D image obtained by the angular refinements is almost indistinguishable from the regular RESIRE with the ground truth angles. At the same time, the two FSC curves are nearly identical (so the figures are omitted here).

### RESIRE reconstruction of the experimental data of a biomineral

To test RESIRE with experimental data, we use a tomographic tilt series acquired from an aragonite coral sample with an annular dark-field (ADF) scanning transmission electron microscope (STEM). The tilt series consists of 69 projections with a tile range from $-60^{\circ }$ to $+76^{\circ }$ and an angular increment of $2^{\circ }$. The projections were pre-processed with background subtraction, normalization, and alignment. Details of the experiment and pre-processing can be found elsewhere^61^.

**Figure 3:**
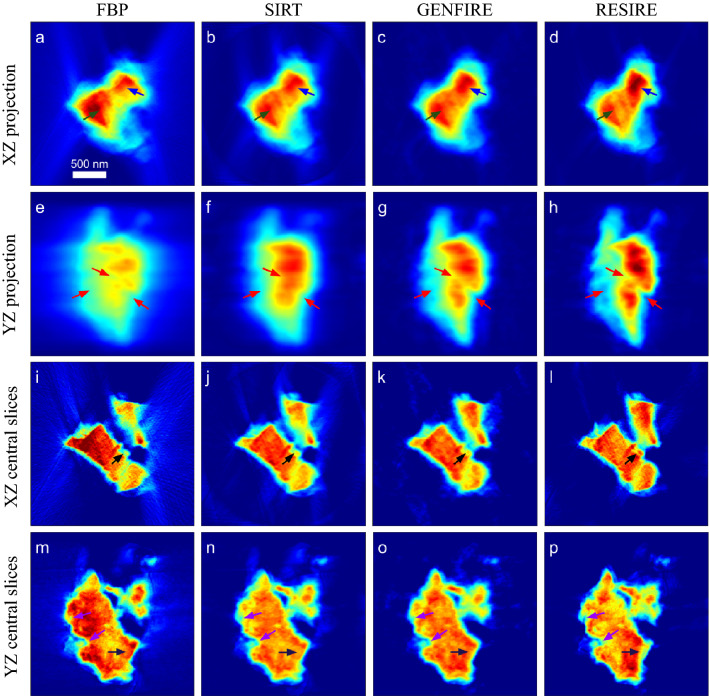
3D reconstruction of an aragonite coral sample. XZ and YZ projections of the FBP (**a, e**), SIRT (**b, f**), GENFIRE (**c, g**) and RESIRE (**d, h**) reconstructions, respectively. 30-nm-thick central slices of the FBP (**i, m**), SIRT (**j, n**), GENFIRE (**k, o**) and RESIRE(**l, p**) reconstructions in the XZ and YZ planes, respectively. The arrows show features that are better reconstructed by RESIRE than by the other three methods. The arrows show that RESIRE produces a reconstruction with less peripheral noise, more reduced missing wedge effects, and sharper fine features than FBP, SIRT and GENFIRE.

Using the same experimental data set, we perform FBP, SIRT, GENFIRE, and RESIRE, reconstructions with $R_F$ of 25.4%, 13.5%, 7.29% and 5.30%, respectively. RESIRE produces the smallest $R_F$, indicating that its 3D reconstruction is most consistent the 69 experimental projections. Figure 3a–h shows the XZ and YZ projections of the four 3D reconstructions, where the z-axis (horizontal) is the missing wedge direction. Compared with FBP, SIRT and GENFIRE, RESIRE reduces the reconstruction artifacts in the missing wedge direction as indicated by arrows. Figure 3i–p shows the 30-nm-thick central slices of the four reconstructions in the XZ, and YZ planes. RESIRE exhibits less peripheral noise, more reduced missing wedge effects, and sharper fine features than the other three methods (arrows).

We also report the computational time of RESIRE in the case of GPU parallel computing. On an intel core i7-7800 computer equipped with a NVIDIA GeForce GTX1080Ti GPU, RESIRE needs 0.15 s per iteration to reconstruct the 3D volume with size $243 \times 243 \times 243$ pixels. Totally, the entire reconstruction accomplishes 200 iterations in 30 s.

### RESIRE reconstruction of the 3D atomic structure of an amorphous film

To perform the tomographic reconstruction of an extended object, we use an amorphous Ta thin film with an average thickness  5 nm, which was fabricated by physical vapor deposition. A tilt series of 46 ADF-STEM projections with a tilt range from $-63.4^{\circ }$ to $+55.5^{\circ }$ were acquired using the TEAM 1 microscope at NCEM and LBNL. The detailed experimental parameters and data pre-processing can be found elsewhere^46^.

**Figure 4:**
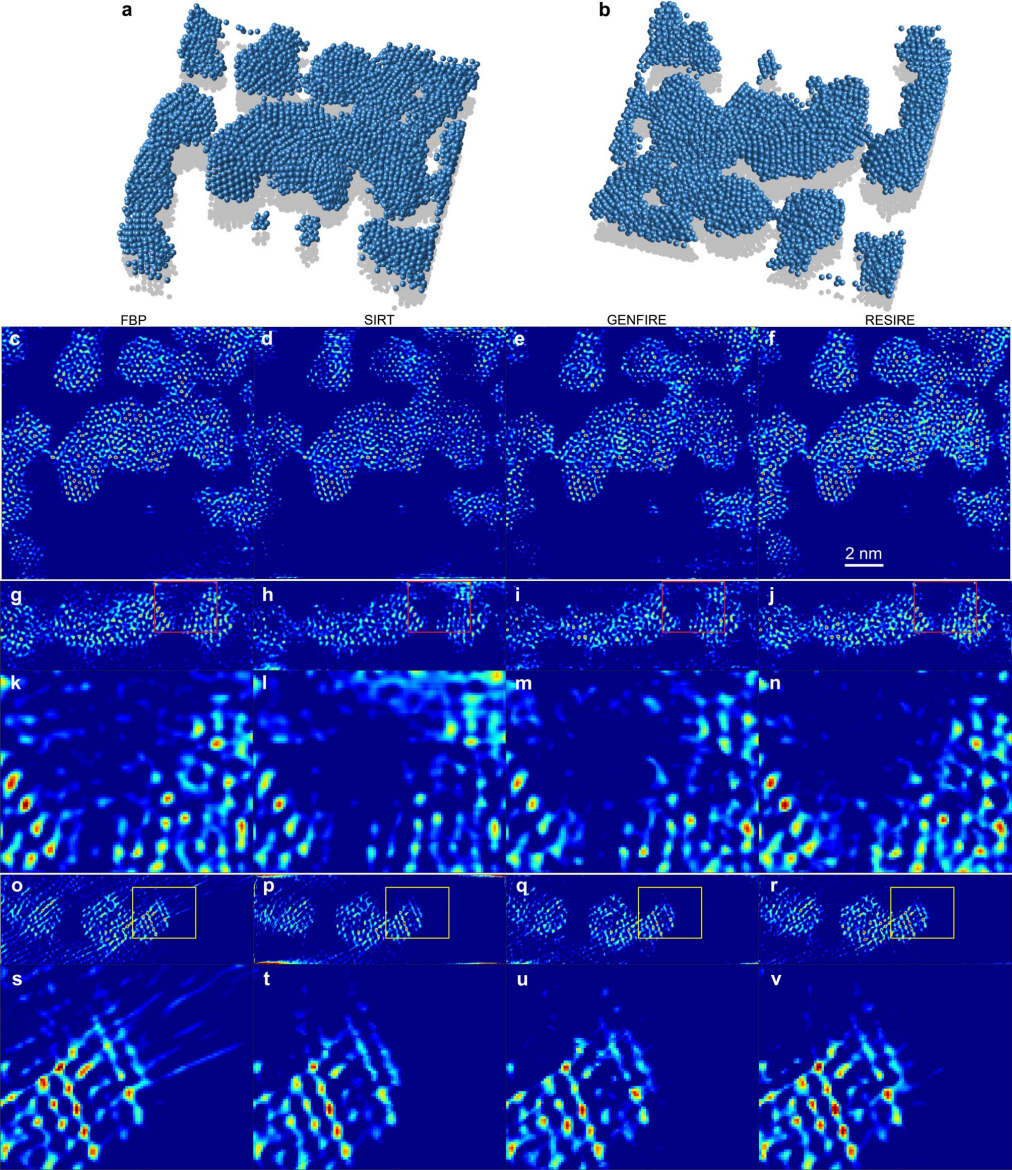
3D reconstruction of an amorphous Ta film at atomic resolution. (**a**–**b**), 3D atomic model of the amorphous Ta thin film at two different orientations, reconstructed by RESIRE. (**c**–**f**), 1.61-Å-thick central slices of the amorphous thin film in the XY plane, reconstructed by FBP, SIRT, GENFIRE and RESIRE, respectively. (**g**–**j**), 1.61-Å-thick central slices in the YZ plane of the reconstructions, where the missing wedge direction is along the z-axis. (**k**–**n**), magnified regions in (**g**–**j**) (red squares), showing that RESIRE produces a high quality reconstruction with least artifacts. (**o**–**r**), 1.61-Å-thick central slices in the XZ plane of the reconstructions. (**s**–**v**), magnified regions in (**o**–**r**) (yellow squares). Compared with the other methods, RESIRE shows less peripheral noise, more easily detectable atomic features and reduces missing wedge effects.

The reconstruction of the amorphous thin film is performed by FBP, SIRT, GENFIRE, and RESIRE. Figure 4a–b shows the 3D atomic model of the amorphous thin film at two different orientations reconstructed by RESIRE, where the atomic tracing procedure is reported elsewhere^46^. Figure 4c–f, shows the 1.61-Å-thick central slices of the amorphous thin film in the XY plane, reconstructed by FBP, SIRT, GENFIRE and RESIRE, respectively, in which RESIRE produces better atomic features than the other methods. Figure 4g–j, (o–r) and their magnified regions (k–n) and (s–v) shows the 1.61-Å-thick central slices in the YZ and XZ planes by the four methods, where more missing wedge effects are clearly visible in the FBP and SIRT reconstructions. Due to the extended nature of the thin film, there are some the artifacts near the top and bottom edges of the slices in the YZ and XZ planes. Compared with FBP, SIRT and GENFIRE, RESIRE exhibits less peripheral noise, more easily detectable atomic features and reduced missing wedge effects.

#### *Remark*

In the first test, we compared the 3D reconstructions of FBP, SIRT, GENFIRE, and RESIRE from 41 projections. In the 3D reconstruction from the experimental data of a biomineral in the second test, we used 69 projections. In the reconstruction of the 3D atomic structure of an amorphous film in the last test, 46 projections were used. The range of the tilt angles is approximately between $-65^{\circ }$ and $65^{\circ }$. In all these cases, RESIRE outperforms other tomographic algorithms. Thus, on the performance of the RESIRE on the projection numbers, we conclude that RESIRE consistently performs more accurate 3D reconstructions than other existing tomographic algorithms when there are a different number of 2D projections.

## Conclusion

We have developed a real-space iterative algorithm, termed RESIRE, for accurate tomographic reconstruction, which uses gradient descent to solve the least-square problem $\Vert \Pi _{\theta }O-b \Vert ^2$. Compared with other real-space iterative algorithms such as ART, SIRT and SART, RESIRE implements a more accurate forward and back projection step in the iterative process. The forward projection step is calculated by either FST with oversampling or Radon transform with sub-voxel precision. The back projection step is computed by a linear transformation. Compared with Fourier-based iterative algorithms such as EST and GENFIRE, RESIRE can be used to reconstruction extended objects in parallel computing. Numerical simulations of a biological vesicle and experimental results of an aragonite coral sample and an amorphous thin film have shown that RESIRE outperforms FBP, SIRT, and GENFIRE with less peripheral noise, more easily detectable boundaries and features, and reduced missing wedge effects. As the power of RESIRE has been demonstrated with the successful reconstructions of amorphous solids and 3D vector ptychography of magnetic materials^45,46^, we expect that RESIRE can be applied to a wide range of imaging modalities in different disciplines, such as AET^1,3,5–10^, coherent diffractive imaging^16–28,62,63^, cryo-electron tomography^14,15^, x-ray absorption and phase contrast imaging^2,42,64–68^, and medical computed tomography^30,34,41^.

## Data Availability

The MATLAB source codes the simulated vesicle data, and the experimental data of the biomineral and the amorphous film are available at https://zenodo.org/record/7273314.
